# Discovery of extracellular vesicles derived miR-181a-5p in patient's serum as an indicator for bone-metastatic prostate cancer

**DOI:** 10.7150/thno.49186

**Published:** 2021-01-01

**Authors:** Yanqing Wang, Yu-Xiang Fang, Baijun Dong, Xinxing Du, Jialin Wang, Xiao Wang, Wei-Qiang Gao, Wei Xue

**Affiliations:** 1 Department of Urology, Renji Hospital, School of Medicine, Shanghai Jiao Tong University, Shanghai 200127, China; 2 State Key Laboratory of Oncogenes and Related Genes, Renji-Med X Clinical Stem Cell Research Center, Renji Hospital, School of Medicine, Shanghai Jiao Tong University, Shanghai 200127, China; 3 School of Biomedical Engineering & Med-X Research Institute, Shanghai Jiao Tong University, Shanghai 200030, China

**Keywords:** prostate cancer, extracellular vesicle, miR-181a-5p, biomarker, bone metastasis

## Abstract

**Purpose:** To identify extracellular vesicle (EV)-delivered microRNAs in the patient's serum as indicators for bone-metastatic prostate cancer.

**Methods:** First, the profiling change of serum EV-delivered miRNAs in patients with either benign prostatic hyperplasia (BPH), non-bone metastatic prostate cancer or bone-metastatic prostate cancer was detected by microRNA deep sequencing assay and microRNA-chip array assay, respectively. Second, the candidates were further confirmed using TaqMan microRNA assay in two independent validation cohorts of total 176 patients with either BPH, non-bone metastatic prostate cancer or bone metastatic prostate cancer to seek the most valuable microRNA(s).

**Results:** Through microRNA deep sequencing and microRNA-chip array, we found 4 prospective EV-delivered miRNAs including miR-181a-5p with significantly upregulated expression in bone metastatic groups than in non-bone metastatic prostate cancer groups (p < 0.05). In the validation cohorts, logistic regression analysis was performed to evaluate the diagnostic association of candidates with bone metastasis, which indicated that miR-181a-5p was significantly associated with bone metastatic prostate cancer. Furthermore, accuracy estimate of each candidate for the diagnosis of bone metastatic prostate cancer was quantified using the area under the receiver-operating characteristic curve (AUC), which identified miR-181a-5p as the best biomarker with the AUCs of 85.6% for diagnosis of prostate cancer and 73.8% for diagnosis of bone metastatic prostate cancer.

**Conclusion:** EV-delivered miR-181a-5p from patient's serum is a promising diagnostic biomarker for bone metastatic prostate cancer.

## Introduction

Prostate cancer is the most common malignancy of the male genitourinary system worldwide 1. In China, the percentage of patients with aggressive or metastatic prostate cancer is relatively higher and the survival rate is significantly lower than that in western countries, which represents a distinct pathologic feature 1-3. Metastasis is a critical and lethal event for prostate cancer patients, along with a dominant termination in the bone. Concomitantly the prostate cancer patients with bone metastasis always develop bone pain or skeletal-related events such as pathologic fractures, spinal cord compression and even myelosuppression 4. Therefore, it is of great demand to detect prostate cancer, especially bone metastatic prostate cancer in Chinese patients as well as in patients worldwide as early as possible.

Up to now, although prostate specific antigen (PSA), a prostate specific, but not a prostate cancer specific expressed gene, is still the most widely used biomarker to screen prostate cancer, it is not an optimal biomarker to early diagnose of prostate cancer as well as its metastasis due to its limited sensitivity and specificity 5, 6. In recent years, accumulating evidence showed that liquid biopsy plays a more and more important role in the early diagnosis of cancers, which can uncover the pathologic characteristics of cancers at molecular levels prior to the tissue biopsy and imaging examination such as magnetic resonance imaging 7, 8. Notably, among the various components contained in the body liquid, extracellular vesicle (EV) is one of the most promising targets to be used in the liquid biopsy 9.

EV is a small vesicle with a diameter of 30-1000 nm, including exosome (30-100 nm diameter) and microvesicle (100-1000 nm diameter), which is generated by all cells and can deliver multiple microRNA, lncRNA, DNA fragments and proteins as cargos enveloped by its lipid bilayer membranes 9, 10. Currently, studies have been reported that EV-delivered microRNAs/proteins can work for information communication and material exchange between tumor cells and surrounding cells (i.e. stromal cells, vascular endothelial cells and immune cells) in the microenvironment to promote tumor progression and to establish a pro-metastatic niche 11-14. Interestingly, it has been found that the profiling change of EV-delivered microRNAs/proteins occurs at a pre-metastatic stage, which indicated an important potential of EV-delivered microRNAs/proteins as biomarkers in the early diagnosis of tumor metastasis by liquid biopsy 15, 16. For example, Costa-Silva et al. found that macrophage migration inhibitory factor was highly expressed in pancreatic ductal adenocarcinomas-derived EVs, indicating a potential biomarker for the development of pancreatic ductal adenocarcinomas liver metastasis 17. In prostate cancer, Alhasan et al. found that several microRNAs (miRNA-200c, miR-605, miR-135a*, miR-433 and miR-106a) enriched in patients' serum EVs were helpful indicators of high-risk prostate cancer 18. In addition, Bhagirath et al. showed that serum EV-delivered miR-1246 was a potential biomarker of aggressive prostate cancer 19. Furthermore, studies from Huang et al. indicated EV-delivered miR-1290 and miR-375 as novel prognostic biomarkers for castration-resistant prostate cancer 20. However, knowledge is limited regarding available EV-delivered microRNAs/proteins as biomarkers for the diagnosis of bone metastatic prostate cancer.

In this study, we attempted to screen and identify novel serum EV-delivered microRNAs for early diagnosis of bone-metastatic prostate cancer. We employed microRNA deep sequencing assay combined with microRNA chip array assay for the primary screen and performed TaqMan microRNA assay for further validation. Through stepwise screen and validation in two independent cohorts, we for the first time demonstrated that serum EV-delivered miR-181a-5p is a potential indicator of bone-metastatic prostate cancer in Chinese patients.

## Materials and Methods

### Clinical samples

This study was approved by the Ethics Committee at the Renji Hospital, Shanghai Jiao Tong University School of Medicine, China. All procedures performed in studies involving human participants were in accordance with the ethical standards of the institutional and with the 1964 Helsinki Declaration and its later amendments or comparable ethical standards. Informed consent was obtained from all individual participants included in the study.

Venous blood samples (5 mL) were collected in non-anticoagulant acquisition tubes (GD050A, Gongdong Comp. Zhejiang, China) for serum extraction (1 mL) one day before prostate biopsy. The coagulation of each sample was carried out at room temperature for 30 min and then the centrifugation (2000 g, 10 min, 4 °C) was performed to extract the serum. All of the serum samples were stored at -80 °C (no more than 6 month) in EP tube till bone metastasis of patients was confirmed by whole body bone scan (Symbia Intevo 16, Siemens) at Renji Hospital. Samples from newly diagnosed patients with untreated bone metastatic prostate cancer were selected in our study. According to general consensus in the clinical practice, the indolent prostate cancer is referred to the prostate cancer with Gleason Score 6, and the aggressive prostate cancer is referred to the prostate cancer with Gleason Score > 6.

### Serum EV extraction and purification

One milliliter serum was used to isolate EV for each sample. After unfrozen in a 25 °C water bath, the serum was centrifuged at 2000 g for 30 min at room temperature to eliminate residual cell fragments. For microRNA deep sequencing assay and microRNA chip array assay, the serum EV was extracted using the Total Exosome Isolation Kit (Thermo fisher scientific) according to the manufacturer's protocol. After EV extraction, we treated samples with RNase (final concentration: 1 μg/mL) as an optimal step to exclude any potential non EV-packed free RNA. Then an exosome Purification and RNA Isolation Kit (Norgen Biotek) was employed to purify EV and extract total RNA in EV consequentially.

### EV quantification

Nanoparticle Tracking Analysis (NTA) was carried out with ZetaView PMX 110 (Particle Metrix, Meerbusch, Germany) and corresponding software ZetaView 8.04.02 for EV quantification. Isolated EV samples were appropriately diluted using 1 X PBS buffer to measure the particle size and concentration at room temperature. NTA measurement was recorded and analyzed at 11 positions randomly. For each position the duration of videos is 40 s. The ZetaView system was calibrated using 110 nm polystyrene particles. All the assays were performed by DKSH Comp. (www.dksh-instrument.cn, Shanghai, China).

### Transmission electron microscopy assay

Morphologies of EV were observed using transmission electron microscopy (TEM). EV sample was resuspended into 50-100 μL 2% paraformaldehyde (PFA) and 5 μL sample suspension was added onto Formvar Carbon network. Samples were fixed on the copper mesh using 50 μL 1% glutaraldehyde. For negative staining of EVs, the copper mesh was incubated on 50 μL uranium oxalate (pH 7) drops for 5 min and in turn on 50 μL methylcellulose droplet for 10 min on ice. After absorbing the excess liquid and air-dry for 10 min, the copper mesh was put in the box for electron microscope photos at 80 kV.

### Western blot

Details of western blot could be found in our previous study 21. Briefly, EV samples were lysed using RIPA buffer (Millipore, Bedford, MA, USA) with Protease Inhibitor Cocktail (PIC, final concentration: 35 μg/mL) and PMSF (Phenylmethanesulfonylfluoride, final concentration: 1 mmol/L) for isolation of total proteins. Protein samples (30 μg, concentration determination by BCA kit from Thermo Fisher Scientific) were separated by 8% SDS-PAGE along with a transfer to the polyvinylidene fluoride membrane (Millipore). After blocking with 5% BSA for 1hr at room temperature, the membrane was incubated with primary antibodies overnight at 4 °C and followed by HRP-conjugated secondary antibody for 1 h at room temperature. After interacting with HRP substrate, protein strips were photographed with the ECL detection apparatus (Thermo Fisher Scientific). Primary antibodies for EV characteristic markers CD9 (1:500), CD63 (1:1,000) and TSG101 (1:1,000), and contaminant markers GM130 (1:1,000), albumin (1:500) and calnexin (1:500) (all from System Biosciences, Palo Alto, CA, https://www.systembio.com) were used for analysis by western blotting.

### MicroRNA deep sequencing assay

Total RNA (10 ng) from serum-derived EVs was extracted and in turn inspected by Qubit2.0 software (Life Technologies, USA) on the Agilent 2200 TapeStation platform (Agilent Technologies, Santa Clara, CA, USA) for sample quality control. Agilent 2100 bioanalyzer electrophoresis system (Agilent Technologies, Santa Clara, CA, USA) was used for quantification of total RNAs. After that, library was constructed and the sequencing was carried out on HiSeq^TM^ 2500 platform according to the user guide using single end (1 × 50) standard sequencing program. The raw data was checked by C++ and R language as a quality control to obtain clean high-quality data. The expression of microRNA was analyzed by Perl software and the differential expression of microRNA was obtained by edgeR software. All the tests and heat map drawing were performed by Ribobio Comp (www.ribobio.com, Guangzhou, Guangdong, China). The raw data of microRNA deep sequencing assay in this study is available in the GEO (Gene Expression Omnibus) database with accession number GSE134205. Quality control results from deep sequencing including numbers of raw reads and mapping reads obtained were listed in Table S1.

### MicroRNA chip array assay

Total RNA from serum-derived EVs was extracted for quality control using Agilent Bioanalyzer 2100 platform (Agilent technologies, Santa Clara, CA, US). After that, miRNA molecular in total RNA was labeled by miRNA Complete Labeling and Hyb Kit (Cat #5190-0456, Agilent technologies, Santa Clara, CA, US) followed the manufacturer's instructions. For array hybridization, each slide was hybridized with 100ng Cy3-labeled RNA in hybridization Oven (Cat #G2545A, Agilent technologies, Santa Clara, CA, US) at 55 °C, 20 rpm for 20 h according to the manufacturer's instructions and then washed with Gene Expression Wash Buffer Kit (Cat #5188-5327, Agilent technologies, Santa Clara, CA, US). EV-delivered total RNAs (10 ng) for each sample were used for microRNA chip array assay by the Agilent Human miRNA Array V16.0 platform. Slides were scanned by Agilent Microarray Scanner (Cat #G2565CA, Agilent technologies, Santa Clara, CA, US) using Feature Extraction software 10.7 (Agilent technologies, Santa Clara, CA, US) with default settings. Raw data were normalized by Quantile algorithm, included in the R package AgiMicroRNA 22. All the tests and heat map drawing were performed by Sangon Biotech Comp (www.sangon.com, Shanghai, China). The raw data of microRNA chip array assay in this study is available in the GEO database with accession number GSE134266. Quality control results from microRNA chip array including normality reads obtained and the detection rates were listed in Table S1.

### MicroRNA extraction and quantitative real-time PCR

For validation of the expression of candidate EV-delivered microRNAs in samples from cohort I (n = 74) and cohort II (n = 102), an Ultra Exol^TM^ Exosome MicroRNA Extraction kit (Cat No. FK-K0102001, Santeja Inc., Japan, for cohort I) and an exoRNeasy kit (Qiagen, Valencia, CA, USA, for cohort II) were used respectively to isolate and purify EV-derived total RNA from serum samples following the manufacturer's instruction. Briefly, EVs were accumulated from serum using spin columns (ASAHI Glass Chemical-based Spin Column, AGC, Japan 23) and then were lysed for subsequent RNA extraction and purification according to a similar protocol contained in these two kits respectively. After that, a *C. elegans* specific microRNA mimic cel-miR-40-3p (100 pM) or cel-miR-54-3p (100 pM, Thermo fisher scientific) was mixed into the samples for subsequent reverse transcription and TaqMan MicroRNA Assay as an exogenous control 18, 19. The qPCR RT-reaction was done by loading the same RNA amount, which was quantified by Nanodrop platform. All candidate EV-delivered microRNAs as well as exogenous control microRNA were specifically reverse transcribed using TaqMan™ MicroRNA Reverse Transcription Kit (Thermo fisher scientific). TaqMan MicroRNA Assay (Thermo fisher scientific) was employed to measure the relative expression of candidate microRNAs, which was neutralized by the exogenous control. The LightCycler480II PCR instrument (Roche) was used to perform the qRT-PCR assay. Delta-delta Ct method was used in the qRT-PCR data analysis. Catalog numbers of reverse transcription primer and microRNA probe set for TaqMan MicroRNA Assay and *C. elegans* specific microRNA mimics were summarized in Table S2.

### Statistics assay

Categorical variables were shown in numbers with proportion (%) and compared by chi-square tests. For continuous variables, independent Student's t-test or analysis of ANOVA was used. Accuracy estimates of each candidate for the presence of prostate cancer, aggressive prostate cancer or bone metastatic prostate cancer were quantified using area under the receiver-operating characteristic curve (AUC). The significance of variance of AUC between candidate microRNA and PSA was analyzed using the Delong method 24. A logistic regression analysis was performed to evaluate the diagnostic association of candidates with bone metastasis by chi-square test. The normality of our data was tested using the MedCalc software (version 15.2.2). All hypothesis tests were two-sided. Results were considered statistically significant when p < 0.05.

## Results

### EV-delivered microRNAs are dysregulated in bone-metastatic prostate cancer

**Figure 1:**
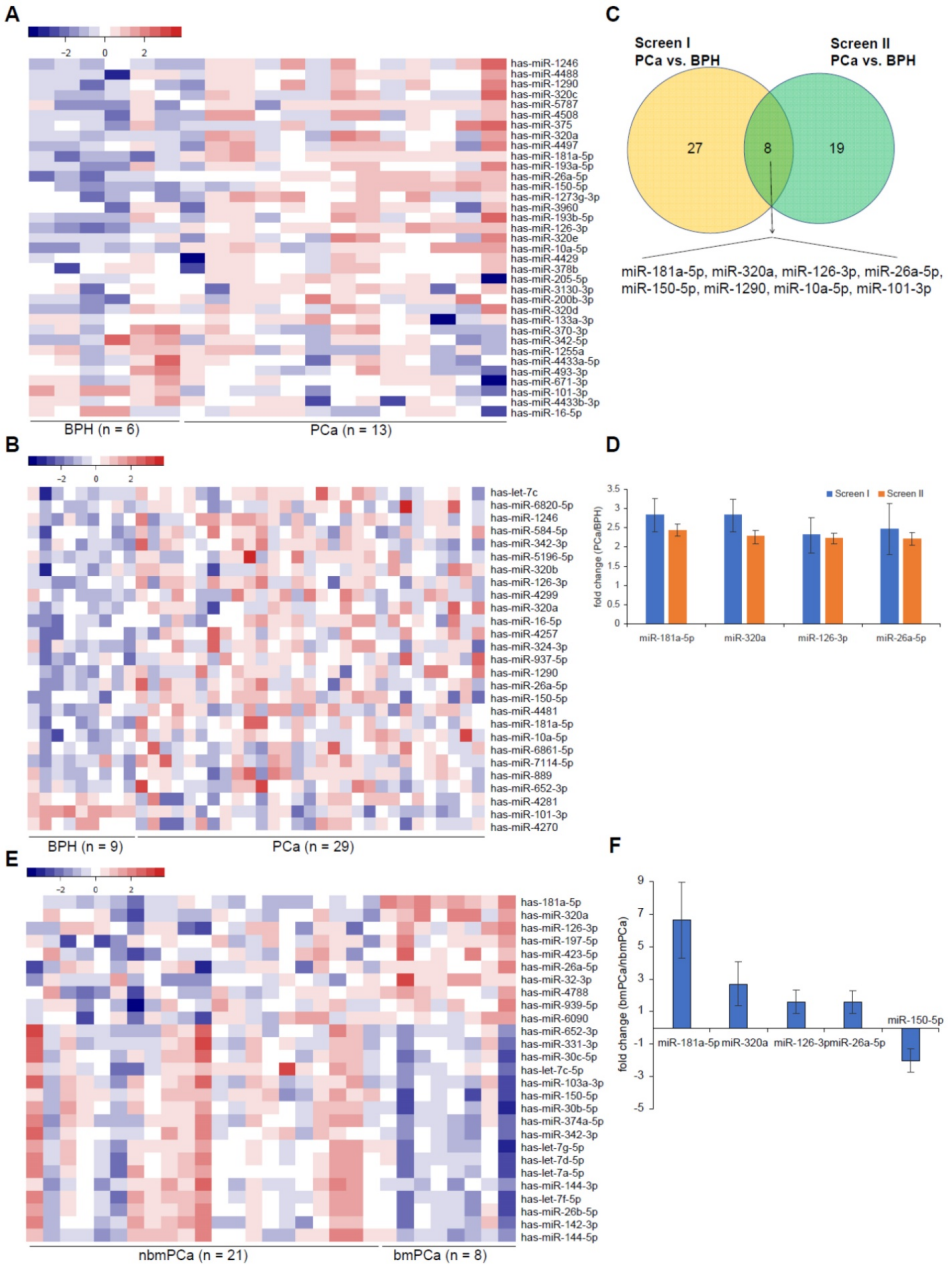
** Screen of differentially expressed EV-delivered microRNAs in PCa vs. BPH and in bmPCa vs. nbmPCa respectively. (A, B)** Heatmap showing differentially expressed EV-delivered microRNAs between BPH and PCa screened by **(A)** microRNA deep sequencing assay or by **(B)** microRNA-chip array assay. **(C)** Venn diagram showing differentially expressed EV-delivered microRNAs between BPH and PCa from both screen I and II. **(D)** Mean fold changes of differentially expressed EV-delivered microRNAs between BPH and PCa. Data were presented by Mean ± SD. **(E)** Heatmap showing differentially expressed EV-delivered microRNAs between nbmPCa and bmPCa screened by microRNA-chip array. **(F)** Mean fold changes of differentially expressed EV-delivered microRNAs between nbmPCa and bmPCa. Data were presented by Mean ± SD. nbmPCa: non-bone metastatic prostate cancer; bmPCa: bone metastatic prostate cancer.

For preliminary screen (Screen I), total RNA was extracted from EVs in serum samples of 6 BPH control cases and 13 prostate cancer patients (including 12 non-bone metastatic prostate cancer patients and 1 bone metastatic prostate cancer patient). The integrity of EV preparations was evaluated by NTA (Figure S1A-C). NTA assay showed that the average EV size (Figure S1B) and concentration (Figure S1C) were no significant difference from BPH group to prostate cancer group. In addition, the expression of two most common EV surface markers CD63 and CD9 as well as the morphological feature of EV by transmission electron microscopy (TEM) observation also exhibited a similar pattern between these two groups (Figure S1D-E). Moreover, we checked the expression of contamination markers albumin, calnexin and Gm130 as well as another EV marker TSG101 in both EVs and tumor tissues by Western Blot as a quality control of EV isolation (Figure S1D). As expected, the expression of three contamination markers was only observed in tissue sample but not in serum derived EVs, indicating a non-contaminative purification of our EV samples. After identification of EVs, RNA was extracted from characterized EVs for profiling assay by microRNA deep sequencing. By PCA plot assay, we found that data from BPH group was focused but data from PCa group was diffused in RNA deep sequencing (Figure S1F). This preliminary screening identified 26 significantly upregulated and 9 significantly downregulated candidate microRNAs in prostate cancer group vs. BPH group (Figure 1A, Figure S2A, Table S3, and Table S4).

Next, we carried out a microRNA-chip array assay in another independent 38 individuals containing 29 prostate cancer and 9 BPH control samples, respectively, to digitally measure the abundance of 2549 common microRNAs, which includes the above 35 dysregulated microRNAs found in microRNA-seq assay. However, by PCA plot assay, we found that data from BPH group did not cluster to distinguish from PCa group. Similarly, data from nbmPCa subgroup did not cluster to distinguish from bmPCa subgroup (Figure S1F). This preliminary screen (Screen II) identified 25 significantly upregulated and 2 significantly downregulated EV-delivered microRNAs (Figure 1B, Table S3 and Table S4). Compared this result to that observed in Screen I, we herein summarized 8 microRNAs (miR-181a-5p, miR-320a, miR-126-3p, miR-26a-5p, miR-1290, miR-10a-5p, miR-101-3p, miR-150-5p), which represented a consistent profiling in both two screens (Figure 1C). Among these microRNAs, 7 of 8 was upregulated 2.4 folds on average in the prostate cancer group in Screen I (from 2.84 ± 0.44 folds for miR-181a-5p to 1.29 ± 0.27 folds for miR-10a-5p) or 2.1 folds on average in the prostate cancer group in Screen II (from 2.44 ± 0.17 folds for miR-181a-5p to 1.32 ± 0.08 folds for miR-10a-5p) when compared to the BPH control group (Figure 1D and Figure S2B). On the other hand, the expression of miR-101-3p was about 2.73 ± 0.36 fold (Screen I) or 2.38 ± 0.09 fold (Screen II) downregulated in the prostate cancer group, respectively (Figure S2B).

Furthermore, we wondered whether all or part of the 8 microRNAs could also work as a biomarker of bone metastatic prostate cancer. To this end, we classified the 29 prostate cancer patients in Screen II into two subgroups according to whether with or without bone metastasis. We investigated microRNA profiling changes between bone metastatic subgroup (n = 8) and non-bone metastatic subgroup (n = 21) using data obtained in Screen II. As results shown, 10 significant upregulated and 17 significant downregulated EV-delivered microRNAs, including 5 of the above 8 candidate microRNAs (i.e. miR-181a-5p, miR-320a, miR-126-3p, miR-26a-5p and miR-150-5p), were revealed in bone metastatic subgroup vs. non-bone metastatic subgroup (Figure 1E and Table S3). By fold change assessment, we found that except for a significant downregulation of miR-150-5p (1.99 ± 0.72 folds, p = 0.0101), all of the other 4 microRNAs showed a significant upregulation for about 3-fold on average in bone metastatic subgroup (6.63 ± 2.32 folds for miR-181a-5p (p < 0.0001), 2.71 ± 1.35 folds for miR-320a (p < 0.0001), 1.62 ± 0.72 folds for miR-126-3p (p = 0.0159), 1.58 ± 0.68 folds for miR-26a-5p (p = 0.0274)), compared to the non-metastatic subgroup (Figure 1F). Considering to the future application of these candidates for early diagnosis via liquid biopsy, we herein focused our further validation on the four upregulated candidate microRNAs. Collectively, these results indicated that these 4 microRNA candidates, especially miR-181a-5p displaying the most significant difference, can become potential markers to distinguish not only between prostate cancer and BPH but also between bone metastatic prostate cancer and non-bone metastatic prostate cancer.

### Validation of EV-delivered microRNAs as biomarkers for prostate cancer and tumor bone metastasis

**Figure 2:**
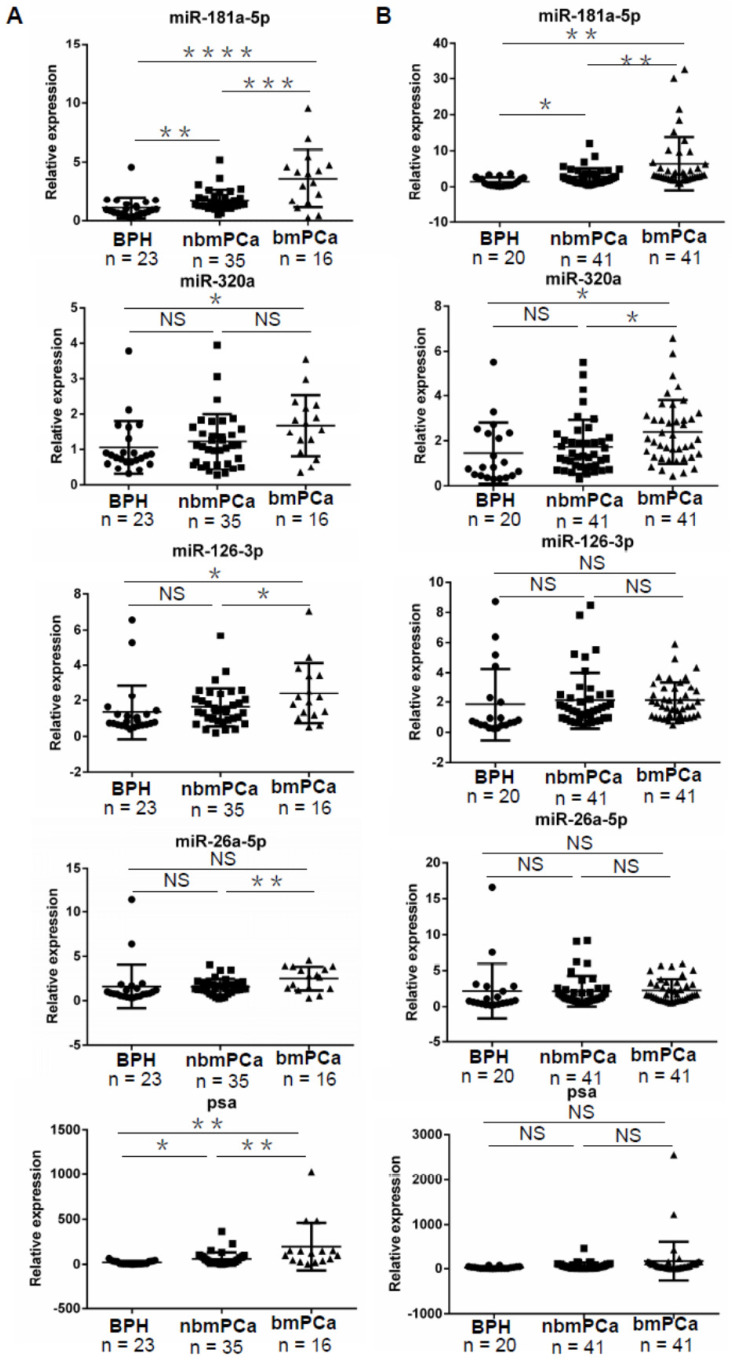
** Validation of differentially expressed EV-delivered microRNAs in BPH, nbmPCa and bmPCa groups. (A)** Relative expression of four differentially expressed EV-delivered microRNAs among BPH controls, nbmPCa patients and bmPCa patients in Cohort I.** (B)** Relative expression of four differentially expressed EV-delivered microRNAs among BPH controls, nbmPCa patients and bmPCa patients in Cohort II. Horizontal lines represent Mean ± SD of data in each group in** (A)** and **(B)**. BPH: Benign prostatic hyperplasia; PCa: prostate cancer; nbmPCa: non-bone metastatic prostate cancer; bmPCa: bone metastatic prostate cancer. The relative expression of each miRNAs was normalized by cel-miR-40-3p. * P < 0.05; ** P < 0.01; *** P < 0.001; **** P < 0.0001; NS: non-significance.

In order to validate our preliminary screen data, we established a cohort (Cohort I) for confirmation of 74 clinical serum samples, including 23 BPH controls, 35 non-bone metastatic prostate cancer (nbmPCa) samples and 16 bone metastatic prostate cancer (bmPCa) samples. The relevant clinical pathologic characteristics were described in Table S3. TaqMan microRNA assay was applied to evaluate the relative expression levels of the 4 candidate microRNAs which were normalized using a *C. elegans* specific microRNA mimic cel-miR-40-3p or cel-miR-54-3p as an exogenous control. We compared the expression of candidate microRNAs in nbmPCa vs. BPH group and bmPCa vs. nbmPCa group respectively. We observed that only the expression of miR-181a-5p but not the other 3 microRNAs exhibited a significant upregulation in nbmPCa (n = 35) vs. BPH group (n = 23) (p < 0.01) and was further increased in bmPCa group (n = 16) (p < 0.001) when using cel-miR-40-3p as an exogenous control, which was consistently with the preliminary screen data (Figure 2A). On the other hand, PSA also showed a significant discrimination among BPH, nbmPCa and bmPCa in this cohort. As a further confirmation, we used another *C. elegans* specific microRNA mimic cel-miR-54-3p as an exogenous control to replace the former cel-miR-40-3p and repeated above validation assays in Cohort I. In this time, we observed that 3 of 4 candidate microRNAs (i.e. miR-181a-5p (p < 0.001), miR-126-3p (p < 0.01) and miR-26a-5p (p < 0.001)) suggested a significant discrimination between BPH and nbmPCa cases (Figure S3A). Thus, these results indicated that the expression of miR-320a was unable to distinguish prostate cancer to BPH whenever which exogenous control was used and was incompetent to act as a biomarker for prostate cancer. Furthermore, when compared the expression of the rest 3 candidate microRNAs in bmPCa vs. nbmPCa, we found that miR-181a-5p kept its significant upregulation in bmPCa group regardless of using cel-miR-40-3p or cel-miR-54-3p as an exogenous control (Figure 2A and Figure S3A). However, both miR-126-3p and miR-26a-5p showed an significant upregulation in bmPCa group when using cel-miR-40-3p as an exogenous control but showed no difference between these two groups when using cel-miR-54-3p as an exogenous control, which indicated that these two microRNAs failed to kept a consistent profiling after control exchange and might not be a stable biomarker for bone metastatic prostate cancer (Figure 2A and Figure S3A).

We next performed another independent and expanded validation assay (Cohort II) with total 102 clinical serum samples, including 20 BPH controls, 41 nbmPCa cases and 41 bmPCa cases, to further identify promising biomarker(s). The relevant clinical pathologic characteristics were described in Table S3. Similar to the data from Cohort I assay, our results again revealed a significant upregulation of miR-181a-5p in nbmPCa cases (n = 41) as compared to BPH controls (n = 20) regardless of using cel-miR-40-3p (p < 0.05) or cel-miR-54-3p (p < 0.05) as an exogenous control (Figure 2B and Figure S3B). However, the rest 3 candidate microRNAs showed no significant difference in nbmPCa vs. BPH cases under the same exogenous control in this Cohort II (Figure 2B and Figure S3B). Notably, the expression of PSA also failed to distinguish nbmPCa vs. BPH cases in this cohort (Figure 2B). For further validation of the potential of miR-181a-5p as bone metastatic biomarkers, we examined the expression levels of miR-181a-5p in bmPCa cases (n = 41) vs. nbmPCa cases (n = 41) in Cohort II. Consistent to previous observation, the expression of miR-181a-5p (p < 0.01) was significantly upregulated in bone metastatic prostate cancer. However, the expression of PSA again showed no significant changes between the two groups, which indicated a limited capability for early diagnosis of bone metastasis (Figure 2B and Figure S3B). Thus, our results indicated that miR-181a-5p was a workable biomarker to discriminate bone metastatic prostate cancer from both non-bone metastatic prostate cancer and BPH.

### EV-delivered miR-181a-5p is a useful biomarker for prostate cancer

**Figure 3:**
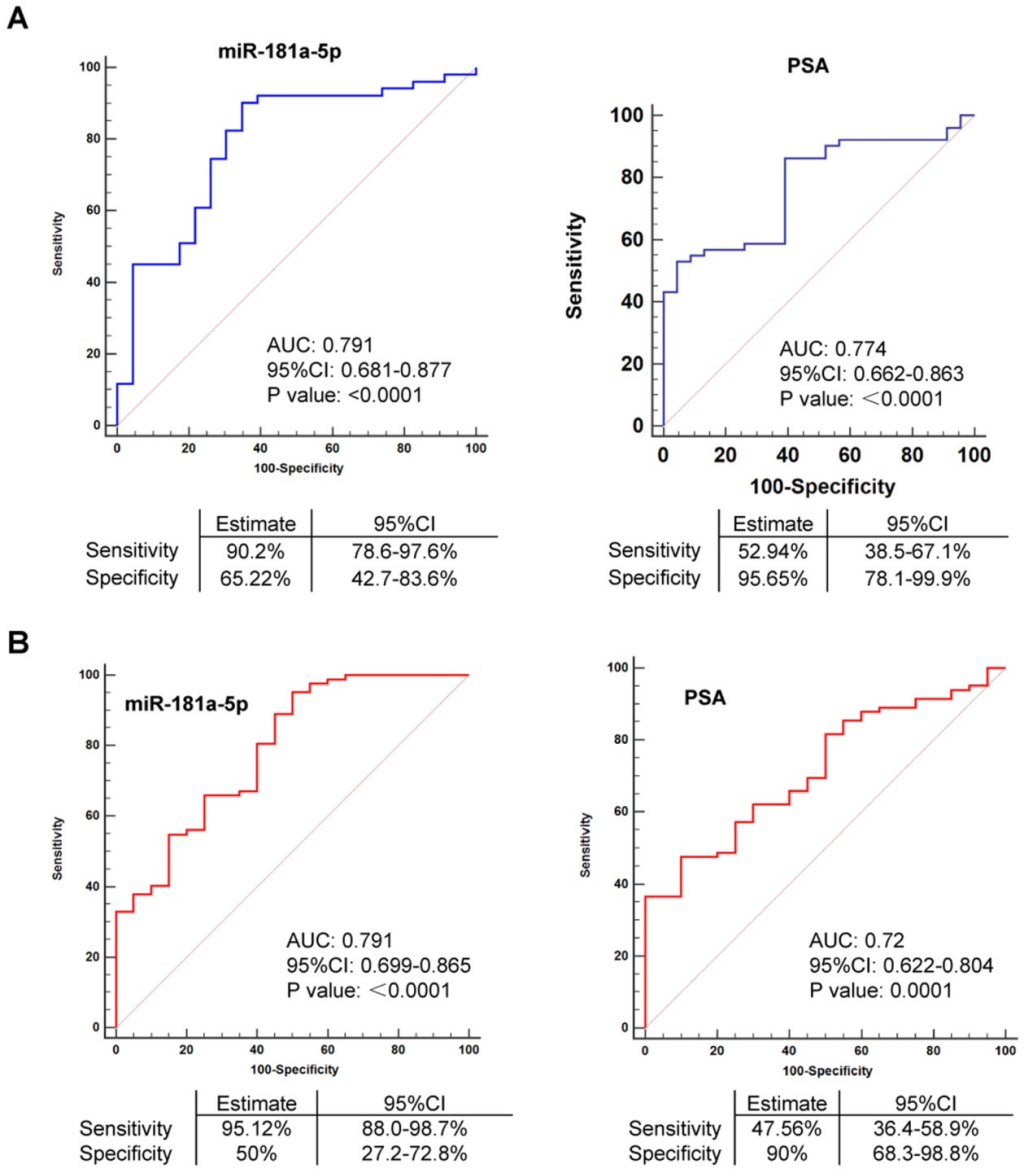
** EV-delivered miR-181a-5p is an available biomarker for PCa. (A)** ROC curve analyses for EV-delivered miR-181a-5p or PSA as a parameter to discriminate PCa from BPH in Cohort I.** (B)** ROC curve analyses for EV-delivered miR-181a-5p or PSA as a parameter to discriminate PCa from BPH in Cohort II. BPH: Benign prostatic hyperplasia; PCa: prostate cancer. The relative expression of each microRNA was normalized by cel-miR-40-3p.

In order to investigate whether miR-181a-5p can be a more effective parameter than PSA to distinguish between BPH and prostate cancer cases, accuracy estimates of each microRNA for the presence of prostate cancer were quantified using AUC assay. In Cohort I, we found that miR-181a-5p showed an AUC of 0.791 (p < 0.0001), 90.2% sensitivity, and 65.22% specificity, when cel-miR-40-3p was used as an exogenous control, but an AUC of 0.856 (p < 0.0001), 88.24% sensitivity, and 82.61% specificity, after the exogenous control was altered to cel-miR-54-3p (Figure 3A and Figure S4A). In addition, PSA showed an AUC of 0.774 (p < 0.0001), 52.94% sensitivity, and 95.65% specificity. By significance assay, the AUC of miR-181a-5p exhibited an equal level to that of PSA (p = 0.776 using cel-miR-40-3p as an exogenous control, p = 0.1898 using cel-miR-54-3p as an exogenous control), indicating that miR-181a-5p can play a similar role to PSA in diagnosis of prostate cancer (Figure 3A and Figure S4A).

Furthermore, we repeated the AUC assay using data in Cohort II. Consistent with the above findings, no significant difference of AUC was observed when either miR-181a-5p or PSA was used as a parameter for diagnosis of prostate cancer (p = 0.1511 using cel-miR-40-3p as an exogenous control, p = 0.0546 using cel-miR-54-3p as an exogenous control) (Figure 3B and Figure S4B). Furthermore, expression of miR-181a-5p was also found to be correlated with clinical parameters of the whole population (Table S5 Part1). Combined with our findings above that the expression of PSA showed no significant difference but miR-181a-5p showed a significant upregulation between BPH and prostate cancer cases in this Cohort II (Figure 2B), our results suggested that EV-delivered miR-181a-5p is a useful biomarker for diagnosis of prostate cancer.

### EV-delivered miR-181a-5p is a potential biomarker for aggressive prostate cancer

**Figure 4:**
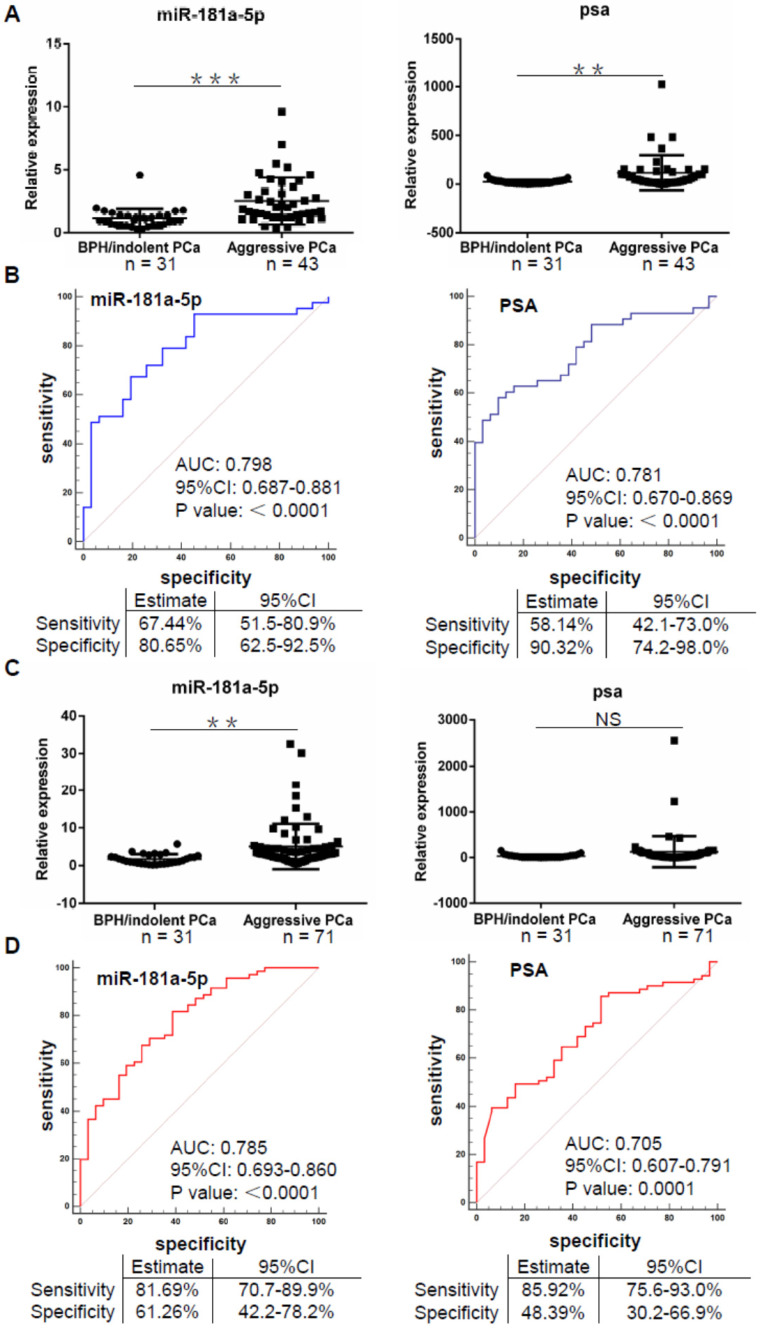
** EV-delivered miR-181a-5p is a potential biomarker for aggressive PCa. (A)** Relative expression of miR-181a-5p in BPH/indolent PCa vs. aggressive PCa in cohort I. Horizontal lines represent Mean ± SD of data in each group.** (B)** ROC curve analyses for miR-181a-5p or PSA as a parameter to discriminate aggressive PCa from BPH/indolent PCa in Cohort I.** (C)** Relative expression of miR-181a-5p in BPH/indolent PCa vs. aggressive PCa in Cohort II. Horizontal lines represent Mean ± SD of data in each group. **(D)** ROC curve analyses for miR-181a-5p or PSA as a parameter to discriminate aggressive PCa from BPH/indolent PCa in Cohort II. BPH: Benign prostatic hyperplasia; PCa: prostate cancer. The relative expression of each microRNA was normalized by cel-miR-40-3p. ** P < 0.01; *** P < 0.001

Up to date, evaluation of aggressive prostate cancer has become an important basis of therapeutic strategy such as active surveillance, radical surgery and endocrine therapy 25. In view of our data indicating a significant upregulation of miR-181a-5p expression in prostate cancer and aggressive prostate cancer cases (Table S5 Part1), we wondered whether EV-delivered microRNAs such as miR-181a-5p can also be used as a biomarker for aggressive prostate cancer. By comparative analysis of microRNA expression data in Cohort I, we found that miR-181a-5p exhibited a significant upregulation in aggressive prostate cancer cases (n = 43) vs. BPH/indolent prostate cancer cases (n = 31) either normalized by cel-miR-40-3p or cel-miR-54-3p (Figure 4A and Figure S4C). By AUC assay, we found that miR-181a-5p showed an AUC of 0.798 (p < 0.0001), 67.44% sensitivity, and 80.65% specificity, when cel-miR-40-3p was used as an exogenous control, but an AUC of 0.84 (p < 0.0001), 93.02% sensitivity, and 70.97% specificity, after the exogenous control was altered to cel-miR-54-3p (Figure 4B and Figure S4D). On the other hand, the expression of PSA was also significantly upregulated in aggressive prostate cancer cases vs. BPH/indolent prostate cancer cases (Figure 4A). In addition, PSA showed an AUC of 0.781 (p < 0.0001), 58.14% sensitivity, and 90.32% specificity (Figure 4B). By significance assay, the AUC of miR-181a-5p exhibited an equal level to that of PSA (p = 0.7962 using cel-miR-40-3p as an exogenous control, p = 0.3133 using cel-miR-54-3p as an exogenous control), indicating that miR-181a-5p can play a similar role to PSA in diagnosis of aggressive prostate cancer.

For further validation in Cohort II, we again observed an upregulated expression of miR-181a-5p in aggressive prostate cancer cases (n = 71) compared to that in BPH/ indolent prostate cancer cases (n = 31) uncorrelated to the exogenous control used (Figure 4C and Figure S4E). However, in this expanded cohort, the expression of PSA showed no significant difference between aggressive prostate cancer cases and BPH/ indolent prostate cancer cases (Figure 4C), which indicated a limitation of PSA on distinguishing these two cases. By AUC assessment, we found that miR-181a-5p showed an AUC of 0.785 (p < 0.0001), 81.69% sensitivity, and 61.26% specificity, when cel-miR-40-3p was used as an exogenous control, but an AUC of 0.824 (p < 0.0001), 73.24% sensitivity, and 80.65% specificity, after the exogenous control was altered to cel-miR-54-3p (Figure 4D and Figure S4F). In addition, PSA showed an AUC of 0.705 (p < 0.0001), 85.92% sensitivity, and 48.39% specificity, which indicated an equal AUC level but a low specificity when compared to miR-181a-5p. Therefore, our findings indicated that EV-delivered miR-181a-5p has a potential to diagnose aggressive prostate cancer as a novel biomarker.

### EV-delivered miR-181a-5p is a promising diagnostic indicator for bone metastatic prostate cancer

**Figure 5:**
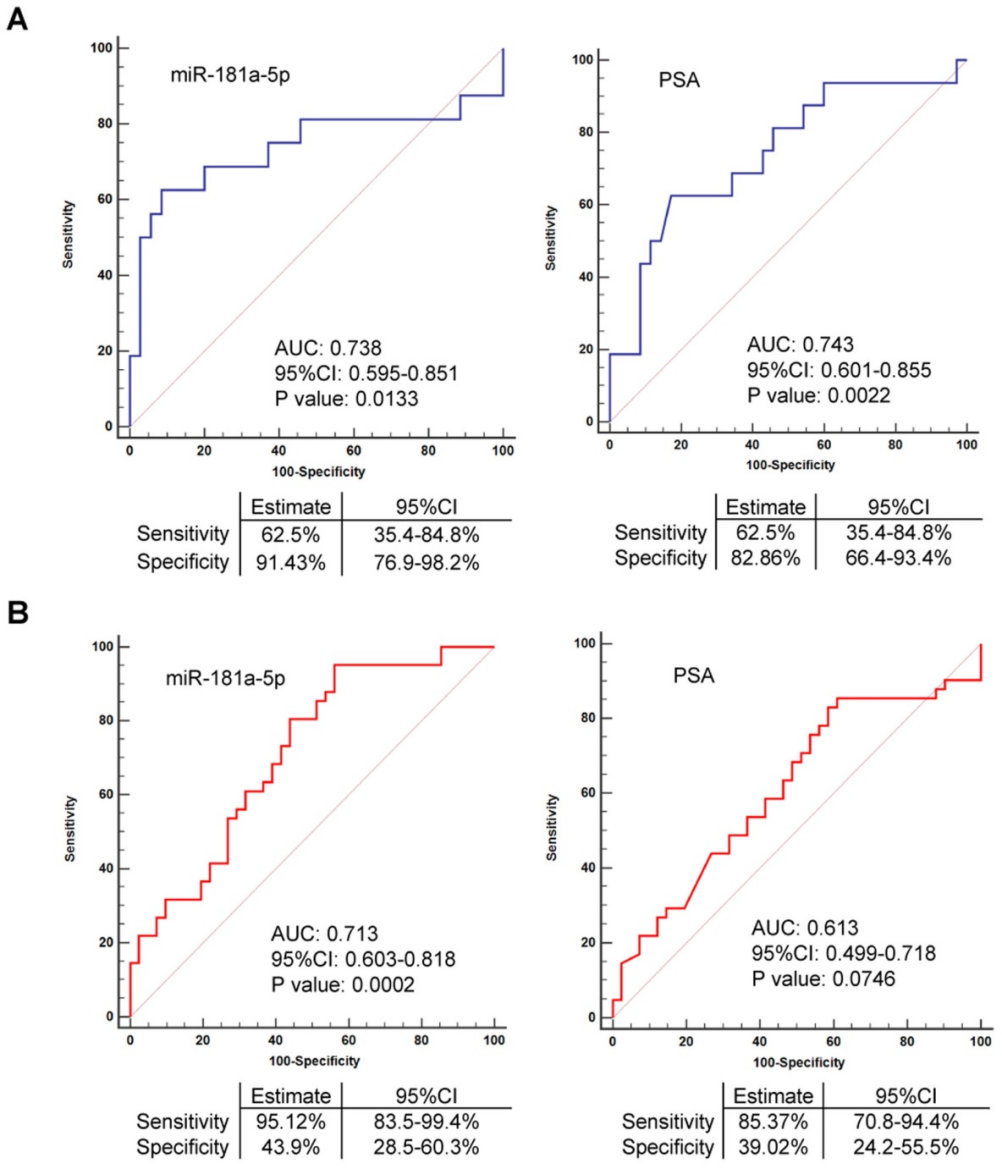
** EV-delivered miR-181a-5p is a promising diagnostic indicator for bone-metastatic PCa. (A)** ROC curve analyses for miR-181a-5p or PSA as a parameter to discriminate bmPCa from nbmPCa in cohort I. **(B)** ROC curve analyses for miR-181a-5p or PSA as a parameter to discriminate bmPCa from nbmPCa in cohort II. nbmPCa: non-bone metastatic prostate cancer; bmPCa: bone metastatic prostate cancer. The relative expression of each microRNA was normalized by cel-miR-40-3p.

As observed in our above validation data, the expression of miR-181a-5p was available to distinguish bone metastatic prostate cancer from non-bone metastatic prostate cancer cases in both Cohort I and Cohort II. In contrast, the expression of PSA was failed to estimate whether the prostate cancer case was a case with or without bone metastasis in the expanded Cohort II (n = 102), although its expression remains a significant difference between these two cases in the relatively small Cohort I (n = 74) (Figure 2A-B). These data indicated miR-181a-5p as a better qualified biomarker than PSA for diagnosis of bone metastatic prostate cancer. As a supporting to this observation, PSA showed an AUC of 0.743 (p = 0.0022) in Cohort I but an AUC of 0.613 (p = 0.0746) in Cohort II (Figure 5A-B). Interestingly, in Cohort I, miR-181a-5p showed an AUC of 0.738 (p = 0.0133), 62.5% sensitivity, and 91.43% specificity, when cel-miR-40-3p was used as an exogenous control, but an AUC of 0.695 (p = 0.0349), 62.5% sensitivity, and 80.0% specificity, after the exogenous control was altered to cel-miR-54-3p (Figure 5A and Figure S4G). In Cohort II, miR-181a-5p showed an AUC of 0.713 (p = 0.0002), 95.12% sensitivity, and 43.9% specificity, when cel-miR-40-3p was used as an exogenous control, but an AUC of 0.719 (p = 0.0001), 87.8% sensitivity, and 53.66% specificity, after the exogenous control was altered to cel-miR-54-3p (Figure 5B and Figure S4H). Thus, these results demonstrated a consistent conclusion, that is, miR-181a-5p worked as a better parameter of bone metastatic prostate cancer. Furthermore, expression of miR-181a-5p was found to be uncorrelated with other clinical parameters of prostate cancer patients except for bone metastasis (Table 1 and Table S5 Part1). By multivariate logistic regression analysis, the expression of miR-181a-5p was also indicated to be significantly associated with bone metastatic prostate cancer, suggesting its independent role for the prediction of bone metastasis (Table 2 and Table S5 Part 2). On the other hand, we checked the intracellular expression of miR-181a-5p by qRT-PCR in tissue samples from patients with BPH (n = 4), nbmPCa (n = 5) and bmPCa (n = 4) respectively. As expected, we observed that expression of miR-181a-5p was upregulated in nbmPCa group vs. BPH control group. Furthermore, we also found an elevated expression of miR-181a-5p in bmPCa group compared to that in nbmPCa group (Figure S5A). In addition, a visual probe (labeled by Cy3) against miR-181a-5p was synthesized and transfected in prostate cancer cell line PC3 and normal prostatic epithelial cell line RWPE-1 to further confirm the expression level of miR-181a-5p. Similarly, we observed an improved expression of miR-181a-5p in PC3 compared to that in RWPE-1 cells (Figure S5B). These data together with our above findings indicated that miR-181a-5p was overexpressed in prostate cancer and could be spread in serum as an EV-delivered microRNA. These data also indicated that EV-delivered miR-181a-5p is a promising diagnostic indicator for bone metastatic prostate cancer.

## Discussion

Although bone metastasis is a main lethal event for prostate cancer patients, specific biomarkers for the early diagnosis of bone metastatic prostate cancer via liquid biopsy have not yet been identified. In this study we for the first time screened and identified an EV-delivered microRNA, miR-181a-5p, as a potential indicator for bone-metastatic prostate cancer in Chinese patients based on high-throughput platforms of deep sequencing and chip array. Importantly, the selective expression of EV-delivered miR-181a-5p was further confirmed using TaqMan microRNA assay in two independent validation cohorts of total 176 patients with either BPH, non-bone metastatic prostate cancer or bone metastatic prostate cancer. Furthermore, logistic regression analysis of the diagnostic association of candidates with bone metastasis indicated that miR-181a-5p was significantly associated with bone metastatic prostate cancer. Using the area under the receiver-operating characteristic curve (AUC), we also showed that miR-181a-5p as the best biomarker among the candidates for diagnosis of tumor bone metastasis.

In view of the more and more extensive application of non-invasive liquid biopsy in early diagnosis of tumorigenesis as well as tumor metastasis, great efforts have been made to identify useful biomarkers for liquid biopsy to evaluate the possibility and the progressiveness of prostate cancer, such as [-2]pro-prostate specific antigen, prostate health index, and prostate cancer antigen 3 26-29. However, their sensitivity and specificity for diagnosis of prostate cancer are not high enough and show large differences among patients from various countries. For example, Wang et al showed that prostate cancer antigen 3 test only moderately improves the diagnostic accuracy in Chinese patients with a PSA of 4.0 - 10.0 ng/mL, but is not superior to %f PSA or PSA density test in patients with a PSA > 10.0 ng/mL 29. On the other hand, although PSA is still used as a golden-standard biomarker to screen prostate cancer and its metastasis, our results found that the expression of PSA was unable to discriminate bone metastatic prostate cancer cases from non-bone metastatic prostate cancer cases after the number of bone metastatic prostate cancer cases was increased from 16 cases in Cohort I to 41 cases in Cohort II (Figure 2A-B). These previous reports and our findings indicate that PSA is not an optimal biomarker to early screen of bone metastatic prostate cancer. Therefore, more accurate and stable biomarkers in the serum or other body liquid are urgently needed for early diagnosis of prostate cancer and particularly of bone metastatic prostate cancer. Accumulating evidence showed that serum EV-delivered cargos (e.g. microRNAs and proteins) can be applied as ideal biomarkers for liquid biopsy in diagnosis of tumor and/or tumor metastasis because of their greater stability under the protection of the vesicle 15, 16, 18, 19. Furthermore, EV-delivered microRNAs have been reported to play an important role in cell-cell communications and be associated with the construction of pro-metastatic niche, which indicated that EV-delivered microRNAs might be promising candidates for early diagnosis of tumor metastasis 30. It is worth mentioned that our results in this study demonstrated that the expression of EV-delivered miR-181a-5p in patient's serum was elevated in non-bone metastatic prostate cancer cases vs. BPH controls (p < 0.05) and was further upregulated in bone metastatic prostate cancer cases (p < 0.01) whenever using cel-miR-40-3p or cel-miR-54-3p as an exogenous control (Figure 2A-B and Figure S3A-B). So that, our current work is in agreement with those other studies, showing that EV-delivered microRNAs from the serum can act as a promising parameter of bone metastatic prostate cancer.

While several microRNAs have previously been reported to be valuable in diagnosis of prostate cancer bone metastasis via profiling assay in prostate cancer tissues in conjunction with metastasis-related data assay *in vivo* 31 and an increased serum level of miR-214 and a decreased serum level of miR-218-5p were indicated to serve as a potential biomarker in prostate cancer patients with bone metastasis, respectively 32, 33, our current work identified a novel, different microRNA compared to the previous studies. We found that EV-delivered miR-181a-5p is a promising diagnostic indicator for bone metastatic prostate cancer with an accuracy of 73.8% in Cohort I and 71.9% in Cohort II, respectively. Support for this conclusion comes from not only our own study, but also reports by others 34-40. First, we performed microRNA deep sequencing assay (n = 19) and microRNA-chip array assay (n = 38) in two independent sample groups to screen candidate microRNAs. By meta-analysis of the data from both assays, we found that 8 microRNAs presented a consistent profiling that significantly discriminates prostate cancer from BPH. Under this condition, 4 of 8 microRNAs were identified to exhibit a significant upregulation in prostate cancer e metastatic prostate cancer vs. non-bone metastatic prostate cancer cases, among which miR-181a-5p showed a most significant upregulation (Figure 1F). Second, two independent validation Cohort I (n = 74) and Cohort II (n = 102) were carried out for validation assay. The major difference of these two cohorts is that more bone metastatic prostate cancer cases are included in Cohort II (with 41 cases) than in Cohort I (with 16 cases). Interestingly, the AUC of miR-181a-5p remained on a certain value when compared the AUC in Cohort I (0.738, p = 0.133) to Cohort II (0.713, p = 0.0002). Unfortunately, the AUC of PSA dropped down from 0.743 in Cohort I (p = 0.0022) to 0.613 in Cohort II (p = 0.0746), which indicate a limitation of PSA on diagnosis of bone metastatic prostate cancer. Notably, we employed two *C. elegans* specific microRNA mimics, cel-miR-40-3p and cel-miR-54-3p, as exogenous controls respectively for validation assay in both cohorts and came to a consistent conclusion, indicating a credible and verifiable application of miR-181a-5p on early diagnosis of bone metastatic prostate cancer although the specificity for miRNA-181-5p in ROC curve analysis was a bit of low in Cohort II due to the limited sample size (Figure 5A-B, Figure S4G-H and Table 2). Therefore, further validation studies from different countries and institutes might contribute to demonstrate the diagnostic function of miRNA-181-5p on bone-metastatic prostate cancer. Third, other groups have reported that miR-181a-5p promotes metastasis in multiple cancers such as breast cancer 34, colorectal cancer 35, 36 and ovarian cancer 37. In prostate cancer, overexpression of miR-181a-5p can promote cancer cell migration and invasion 38, and the expression of miR-181a-5p is upregulated in samples of metastatic prostate cancer when compared to primary prostate cancer 39. Furthermore, the expression of EV-delivered miR-181a-5p was significantly improved under a hypoxia condition 40. Combined these other groups' studies with our findings, we can get a hint that the expression of miR-181a-5p might be enhanced in prostate cancer cells not only to promote cancer cell proliferation and migration by intracellular overexpression but also to increase an EV-delivered secretion to the environment for prostate cancer metastasis. Nevertheless, the cell origin of miR-181a-5p and the mechanism of miR-181a-5p on promotion of metastasis in vivo are needed to be further investigated. Taking together, these expressional profiling assay data in clinical samples together with in vitro mechanism researches on regulation of metastasis by miR-181a-5p reinforce the notion that EV-delivered miR-181a-5p can act as a novel diagnostic biomarker of bone metastatic prostate cancer.

## Conclusion

Our finding revealed that EV-delivered miR-181a-5p from the serum of prostate cancer patients is a promising novel biomarker for early diagnosis of bone metastatic prostate cancer. An extended validation cohort containing patients from China as well as other Asian and Western countries with an increased sample size might be helpful to further consolidate our finding.

## Supplementary Material

Supplementary figures.

Supplementary table 1.

Supplementary table 2.

Supplementary table 3.

Supplementary table 4.

Supplementary table 5.

## Figures and Tables

**Table 1:** Correlation of serum EV-delivered miR-181a-5p expression with clinical pathologic parameters in prostate cancer patients in Cohort I + II (n=133)

Parameters	Total (n, %)	Relative EV derived miR-181a expression*			P-value
		High (> 1.25)	No change (0.75 - 1.25)	Low (< 0.75)	
Age, years					P = 0.623
<60	13 (9.8)	12 (92.3)	0 (0)	1 (7.7)	
60-69	50 (37.6)	39 (78)	9 (18)	2 (4)	
70-79	56 (42.1)	44 (78.6)	8 (14.3)	4 (7.1)	
>79	14 (10.5)	11 (78.6)	3 (21.4)	0 (0)	
PSA, μg/L					P = 0.252
<10	21 (15.8)	15 (71.4)	5 (23.8)	1 (4.8)	
10-19.99	19 (14.3)	12 (63.2)	6 (31.6)	1 (5.3)	
20-49.99	29 (21.8)	23 (79.3)	4 (13.8)	2 (6.9)	
>49.99	64 (48.1)	56 (87.5)	5 (7.8)	3 (4.7)	
Gleason score					P = 0.037
6	20 (15)	12 (60)	7 (35)	1 (5)	
3+4	9 (6.8)	5 (55.6)	4 (44.4)	0 (0)	
4+3	33 (24.8)	27 (81.8)	4 (12.1)	2 (6.1)	
8	38 (28.6)	33 (86.8)	3 (7.9)	2 (5.3)	
9-10	33 (24.8)	29 (87.9)	2 (6.1)	2 (6.1)	
T stage					P = 0.061
T2a	11 (8.3)	6 (54.5)	5 (45.5)	0 (0)	
T2b	17 (12.8)	11 (64.7)	5 (29.4)	1 (5.9)	
T2c	53 (39.8)	45 (84.9)	6 (11.3)	2 (3.8)	
T3	22 (16.5)	19 (86.4)	2 (9.1)	1 (4.5)	
T4	30 (22.6)	25 (83.3)	2 (6.7)	3 (10)	
Risk stratification					P = 0.096
Low	4 (3)	2 (50)	2 (50)	0 (0)	
Intermediate	14 (10.5)	9 (64.3)	4 (28.6)	1 (7.1)	
High/Locally advanced/Metastatic	115 (86.5)	95 (82.6)	14 (12.2)	6 (5.2)	
Lymph node metastasis					P = 0.243
No	95 (73.27)	74 (91.89)	17 (8.11)	4 (0)	
Yes	38 (26.73)	32 (92.58)	3 (3.71)	3 (3.71)	
Bone metastasis					P = 0.009
No	76 (57.1)	54 (71.1)	17 (22.4)	5 (6.6)	
Yes	57 (42.9)	52 (91.2)	3 (5.3)	2 (3.5)	

*: Normality with cel-miR-40-3p as an exogenous control

**Table 2:** Multivariate analyses of the association of predictors with prostate cancer, aggressive prostate cancer or bone metastatic prostate cancer*

Parameters	Prostate cancer		Aggressive prostate cancer		Bone-metastatic prostate cancer	
	OR (95%CI)	P value	OR (95%CI)	P value	OR (95%CI)	P value
Age, years	-	0.181	-	0.620	-	-
PSA, μg/L	-	0.064	1.015 (1.005-1.025)	0.004	1.006 (1.001-1.010)	0.011
Relative expression of EV derived miR-181a	2.506 (1.658-3.789)	<0.001	1.962 (1.373-2.804)	<0.001	1.395 (1.155-1.685)	0.001

*: Normality with cel-miR-40-3p as an exogenous control

## Abbreviations

AUC: receiver-operating characteristic curve
bmPCa: bone metastatic prostate cancer
BPH: benign prostatic hyperplasia
EV: extracellular vesicle
nbmPCa: non-bone metastatic prostate cancer
NTA: nanoparticle tracking analysis
PSA: prostate specific antigen
