# Supplementary material for: Discovery of extracellular vesicles derived miR-181a-5p in patient's serum as an indicator for bone-metastatic prostate cancer
Source: Theranostics. 2021 Jan 1;11(2):878–92. doi: 10.7150/thno.49186 (PMC7738844; doi:10.7150/thno.49186)
Supplement: Supplementary file 1 — Supplementary figures. [file thnov11p0878s1.pdf]

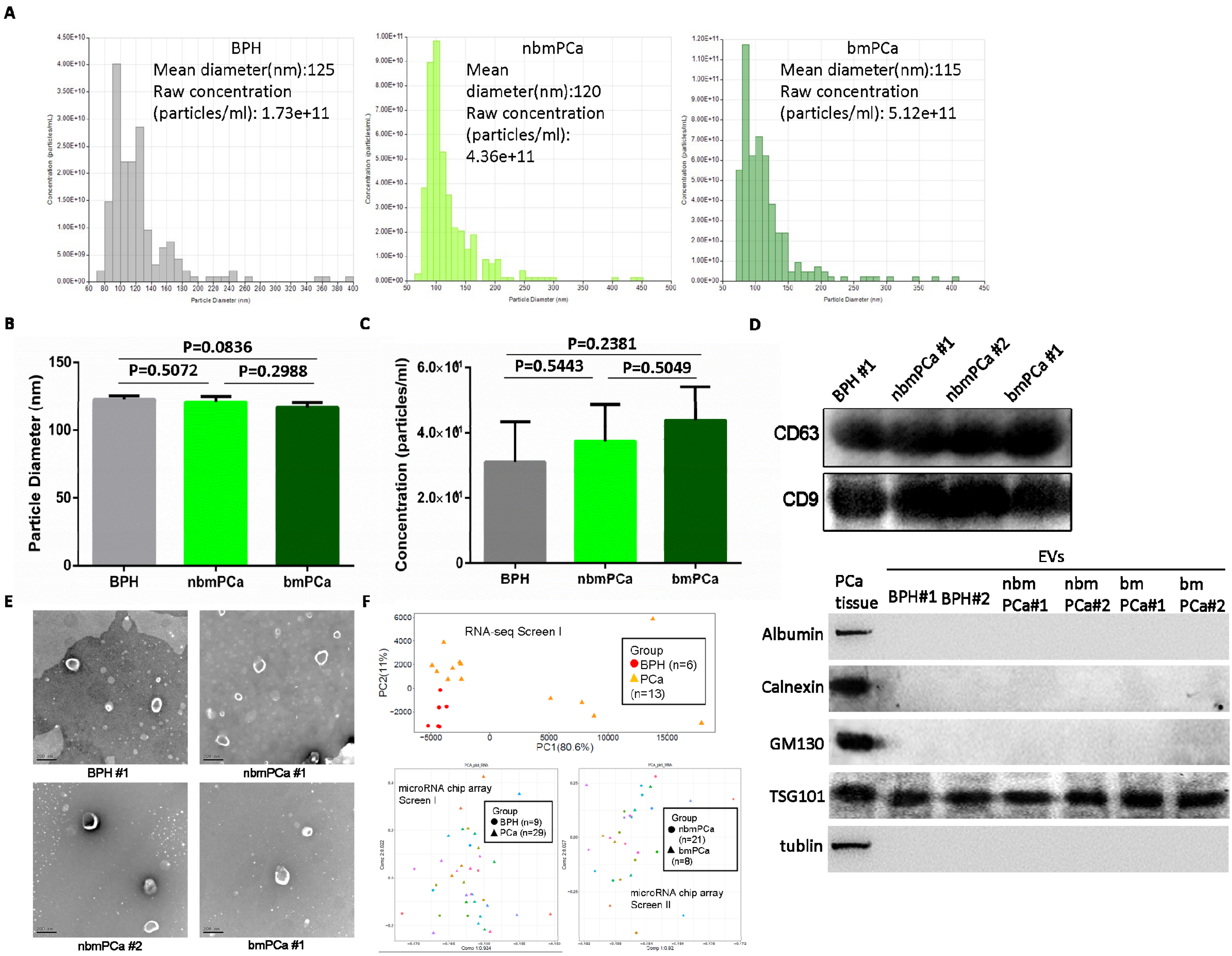

Figure S1. Characterization of serum derived EV preparations. (A) The integrity of serum derived EV preparations is confirmed by NTA. Data from one of the BPH, nbmPCa or bmPCa cases are showed as representative example respectively. **(B)** The comparison of diameter of serum derived EV among BPH, nbmPCa and bmPCa group. Data were presented by Mean ± SD. **(C)** The comparison of concentration of serum derived EV among BPH, nbmPCa and bmPCa group. Data were presented by Mean ± SD. **(D)** The expression of EV markers and contamination markers is detected among BPH, nbmPCa and bmPCa group by western blot. **(E)** The comparison of EV feature among BPH, nbmPCa and bmPCa group by transmission electron microscopy (TEM) assay. Scale Bar: 200nm. **(F)** The cluster of different groups in RNA-seq or microRNA chip array is analyzed by PCA plot assay. BPH: Benign prostatic hyperplasia; non-bone metastatic prostate cancer; bmPCa: bone metastatic prostate cancer.

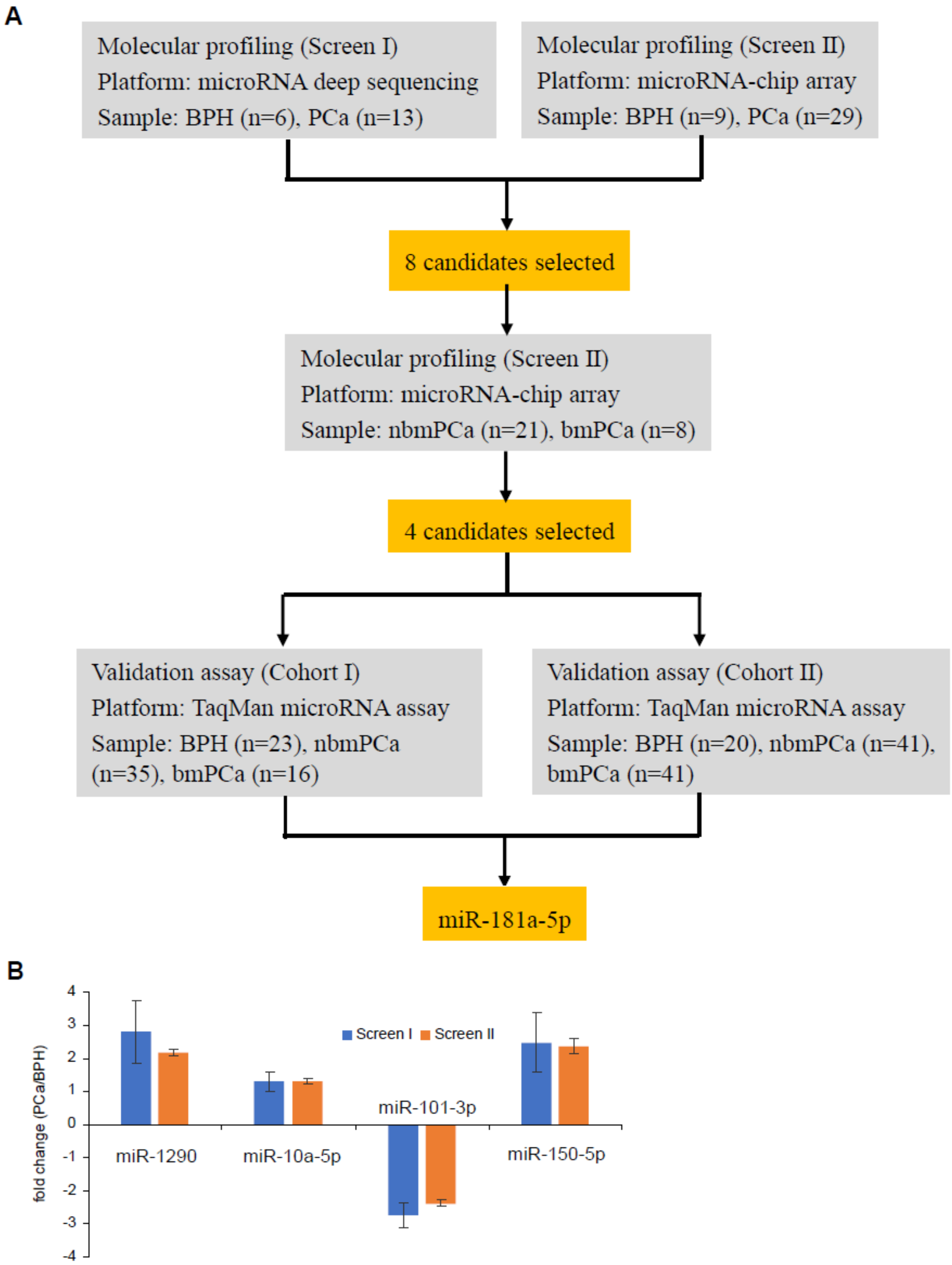

Figure S2. Stepwise screen and validation of EV-delivered microRNAs for early diagnosis of bone-metastatic PCa. (A) Flowchart for the stepwise screen and validation of EV-delivered microRNAs for early diagnosis of bone-metastatic PCa. **(B)** Mean fold changes of differentially expressed EV-delivered microRNAs between BPH and PCa. Data were presented by Mean ± SD.

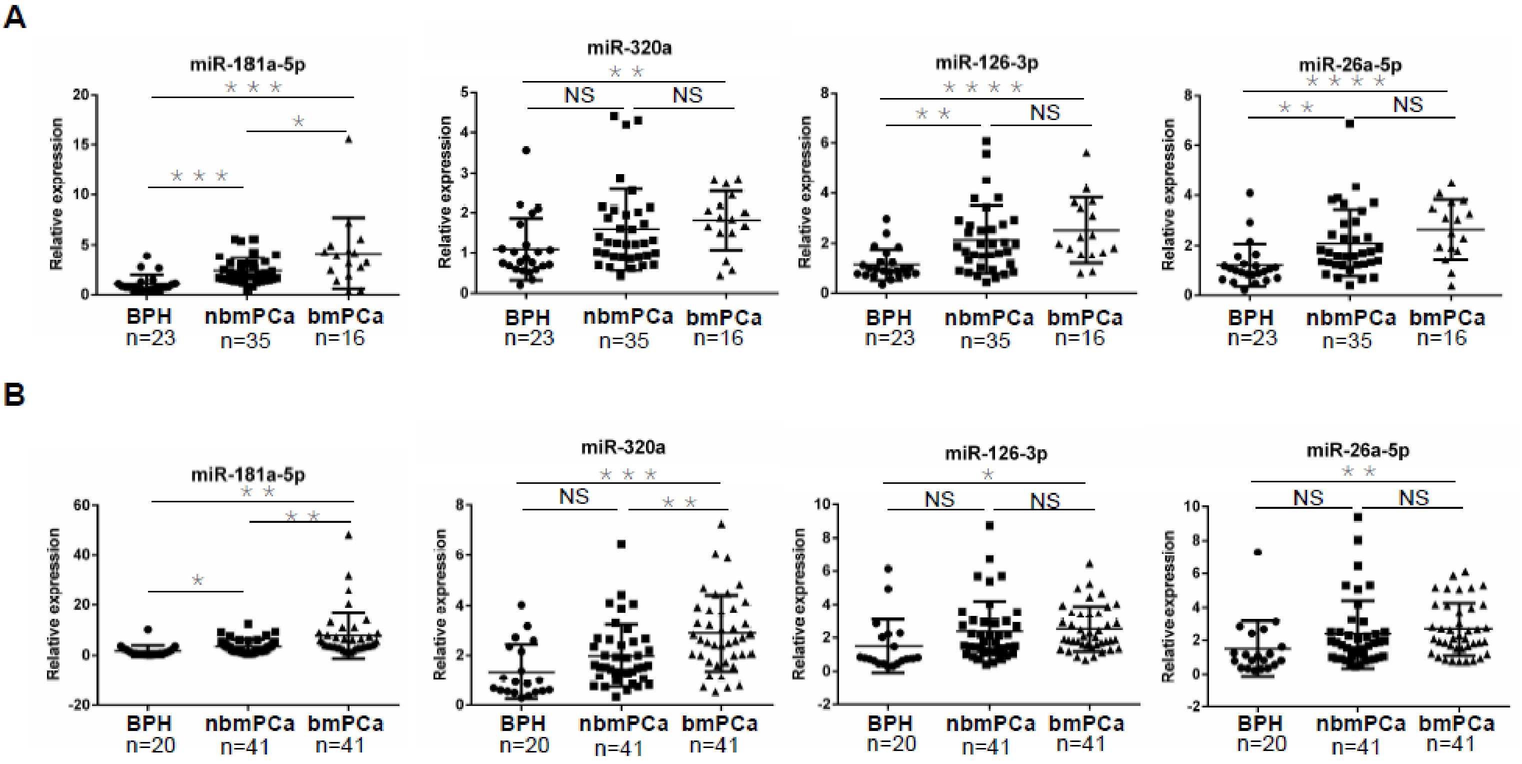

Figure S3. Validation of differentially expressed EV-delivered microRNAs in BPH, nbmPCa and bmPCa groups. (A) Relative expression of four differentially expressed EV-delivered microRNAs among BPH controls, nbmPCa patients and bmPCa patients in Cohort I. **(B)** Relative expression of four differentially expressed EV-delivered microRNAs among BPH controls, nbmPCa patients and bmPCa patients in Cohort II. Horizontal lines represent Mean ± SD of data in each group in **(A)** and **(B)**. BPH: Benign prostatic hyperplasia; PCa: prostate cancer; nbmPCa: non-bone metastatic prostate cancer; bmPCa: bone metastatic prostate cancer. The relative expression of each miRNAs was normalized by cel-miR-54-3p. * P<0.05; ** P<0.01; *** P<0.001; **** P<0.0001; NS: nonsignificance.

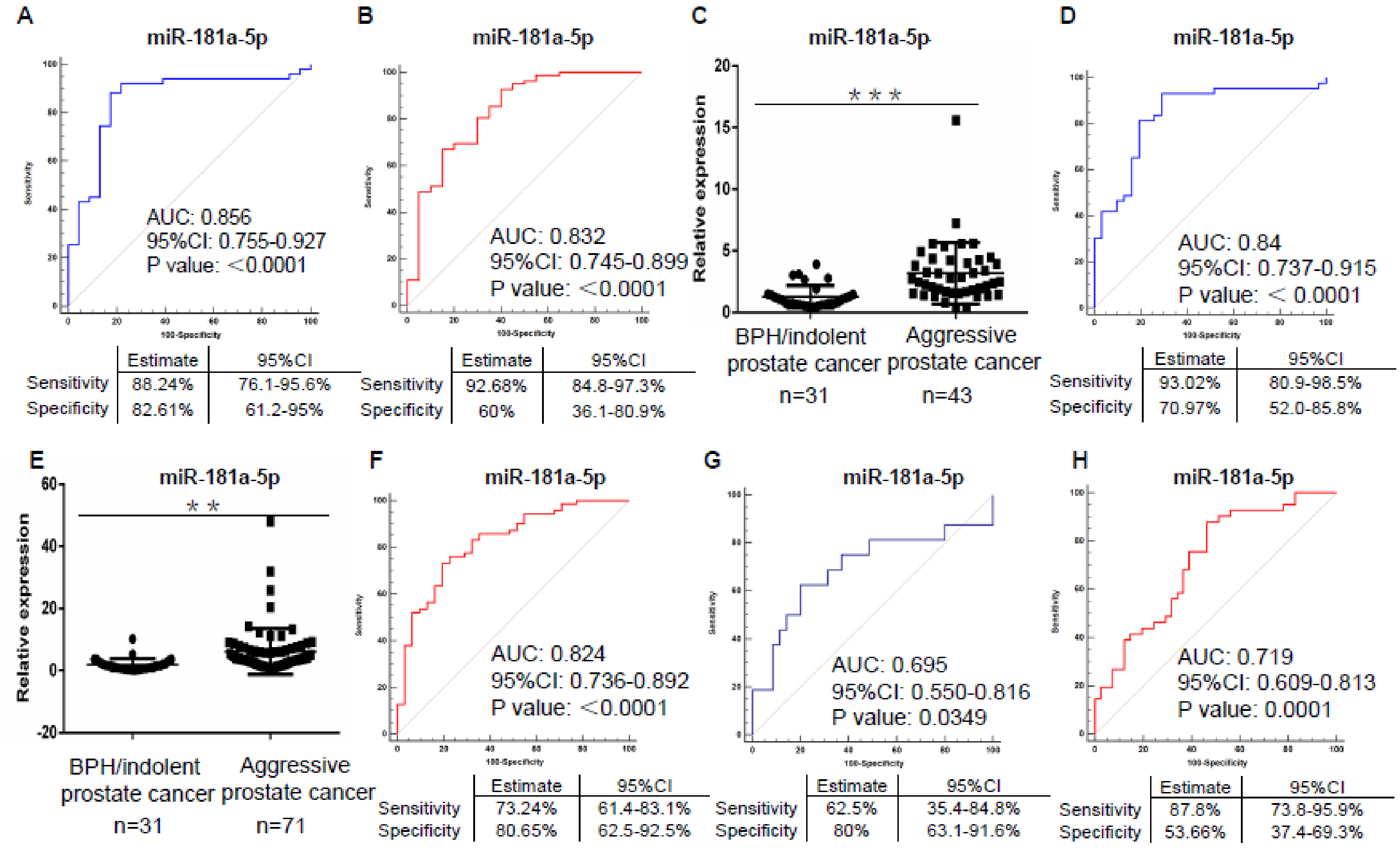

Figure S4. EV-delivered miR-181a-5p is an available biomarker for prostate cancer, aggressive prostate cancer and bone-metastatic prostate cancer. (A, B) ROC curve analyses for EV-delivered miR-181a-5p as a parameter to discriminate prostate cancer from BPH in Cohort I and II. **(C, E)** Relative expression of miR-181a-5p in BPH/indolent prostate cancer vs. aggressive prostate cancer in cohort I and II. Horizontal lines represent Mean ± SD of data in each group in **(C)** and **(E)**. **(D, F)** ROC curve analyses for miR-181a-5p as a parameter to discriminate aggressive prostate cancer from BPH/indolent prostate cancer in Cohort I and II. **(G, H)** ROC curve analyses for EV-delivered miR-181a-5p as a parameter to discriminate bmPCa from nbmPCa in Cohort I and II. BPH: Benign prostatic hyperplasia; nbmPCa: non-bone metastatic prostate cancer; bmPCa: bone metastatic prostate cancer. The relative expression of each microRNA was normalized by cel-miR-54-3p. ** P<0.01; *** P<0.001

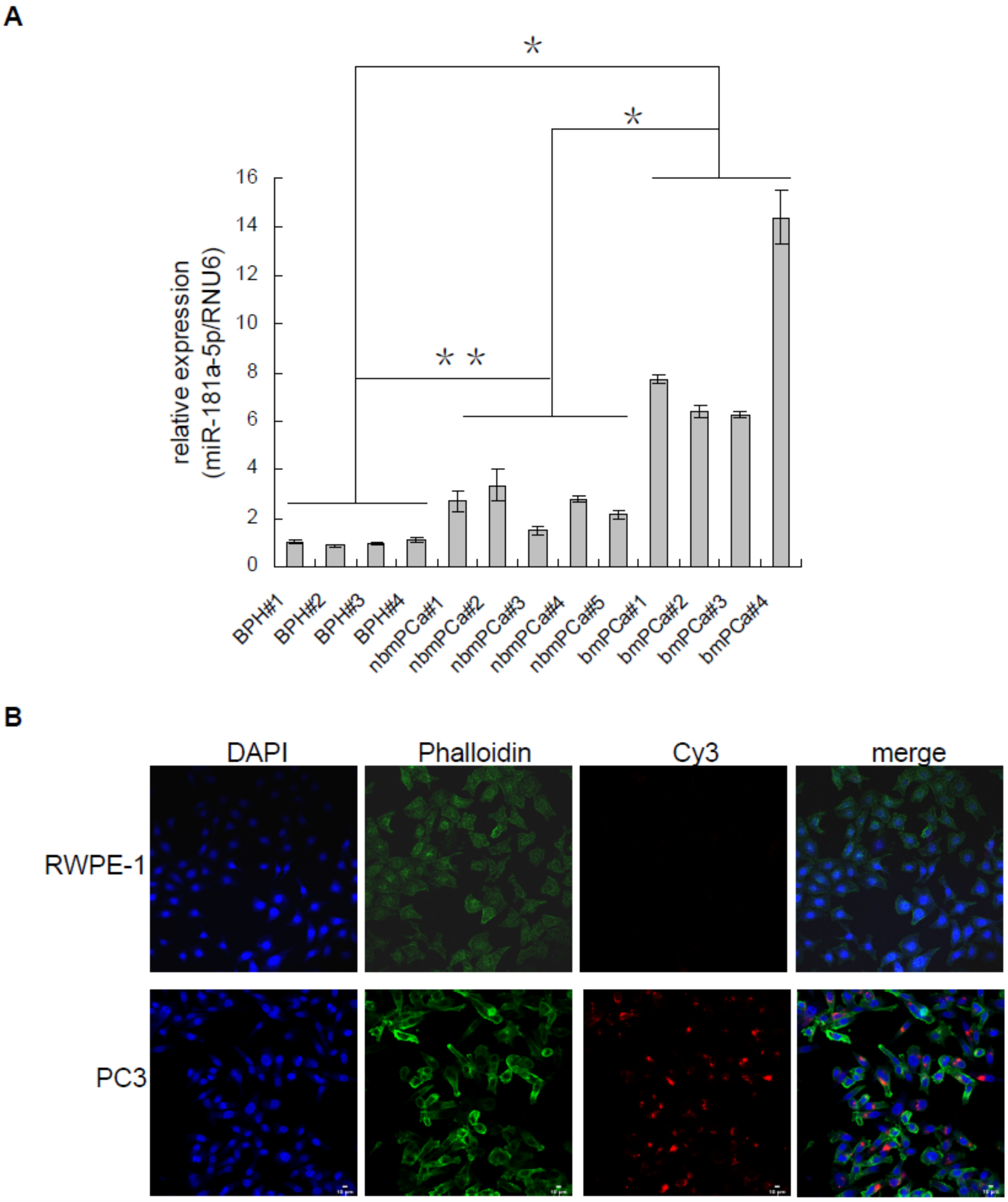

Figure S5. miR-181a-5p is overexpressed in PCa tissues. (A) Relative expression of miR-181a-5p in tissues from BPH controls, nbmPCa patients or bmPCa patients by qRTPCR. Data was presented by Mean ± SD. **(B)** MiR-181a-5p is overexpressed in the prostate cancer cell line PC3 when compared to the normal prostatic epthelial cell line RWPE-1 by transfecting a Cy3-labeled miR-181a-5p probe. BPH: Benign prostatic hyperplasia; PCa: prostate cancer; nbmPCa: non-bone metastatic prostate cancer; bmPCa: bone metastatic prostate cancer. Scale Bar: 10μm, * $P<0.05$; ** $P<0.01$.
