# Supplementary material for: Discovery of extracellular vesicles derived miR-181a-5p in patient's serum as an indicator for bone-metastatic prostate cancer
Source: Theranostics. 2021 Jan 1;11(2):878–92. doi: 10.7150/thno.49186 (PMC7738844; doi:10.7150/thno.49186)
Supplement: Supplementary file 6 — Supplementary table 5. [file thnov11p0878s6.pdf]

Table S5 Part1. Correlation of serum EV-delivered miR-181a-5p expression with clinical pathologic parameters in whole population in Cohort I + II (n=176)

**Parameters**	**Total (n, %)**	**Relative EVderived miR-181a expression***			**P-value**
		High (> 1.25)	No change (0.75 - 1.25)	Low (< 0.75)	
**Age, years**					
**<60**	22（12.5）	14（63.6）	1（4.5）	7（31.8）	P = 0.055
**60-69**	69（39.2）	45（65.2）	14（20.3）	10（14.5）	
**70-79**	69（39.2）	49（71.0）	12（17.4）	8（11.6）	
**>79**	16（9.1）	13（81.3）	3（18.8）	0（0）	
**PSA, µg/L**					
**<10**	42（23.9）	16（38.1）	10（23.8）	16（38.1）	P < 0.0001
**10-19.99**	23（13.1）	13（56.5）	7（30.4）	3（13）	
**20-49.99**	44（25）	33（75）	8（18.2）	3（6.8）	
**>49.99**	67（38.1）	59（88.1）	5（7.5）	3（4.5）	
**Pathological results1**					
**Non-Prostate cancer**	43（24.4）	15（34.9）	10（23.3）	18（41.9）	P < 0.0001
**prostate cancer**	133(75.6）	106（79.7）	20（15）	7（5.3）	
**Pathological results2**					
**BPH / indolent prostate cancer**	62（35.2）	26（41.9）	17（27.4）	19（30.6）	P < 0.0001
**aggressive prostate cancer**	114(64.8）	95（83.3）	13（11.4）	6（5.3）	

*: Normality with cel-miR-54-3p as an exogenous control

Table S5 Part2: Univariate analyses of the association of predictors with prostate cancer, aggressive prostate cancer or bone metastatic prostate cancer*

**Parameters**	prostate cancer		Aggressive prostate cancer		Bone-metastatic prostate cancer	
	OR (95%CI)	P value	OR (95%CI)	P value	OR (95%CI)	P value
**Age, years**	1.060 (1.016-1.107)	0.007	1.054 (1.014-1.096)	0.008	1.013 (0.970-1.058)	0.564
**PSA, µg/L**	1.027 (1.013-1.041)	<0.001	1.022 (1.012-1.032)	<0.001	1.005 (1.001-1.009)	0.027
**Relative expression of EV derived miR-181a**	2.506 (1.658-3.789)	<0.001	2.262 (1.623-3.154)	<0.001	1.401 (1.154-1.701)	0.001

*: Normality with cel-miR-54-3p as an exogenous control
